# Signers and Speakers Show Distinct Temporal Kinematic Signatures in Their Manual Communicative Movements

**DOI:** 10.1162/opmi.a.18

**Published:** 2025-08-29

**Authors:** Rui Liu (刘睿), Wim Pouw, Susan Goldin-Meadow, Diane Brentari

**Affiliations:** Donders Institute for Brain, Cognition, and Behaviour, Radboud University, Nijmegen, The Netherlands; Department of Experimental Psychology, Ghent University, Ghent, Belgium; Department of Cognitive Science and AI, Tilburg University, Tilburg, The Netherlands; Departments of Psychology and Comparative Human Development, University of Chicago, Chicago, IL, USA; Department of Linguistics, University of Chicago, Chicago, IL, USA

**Keywords:** ASL, sign language, hand gesture, motion tracking, kinematic analysis

## Abstract

Using our hands to move a stick along a path differs in systematic ways from using our hands to communicate about moving the stick. Kinematic signatures (e.g., enlarged moving trajectories) have been found to mark a movement as communicative, relative to its non-communicative counterpart. But communicative movements are frequently embedded within an expressive system and might differ as a function of that system. For example, deaf signers move their hands when they communicate with sign language, which is a linguistic system. Hearing speakers also move their hands—they gesture along with speech—but those gestures do not form a linguistic system unto themselves. Do the communicative movements signers and speakers use to describe the same event differ as a function of the expressive systems within which they are embedded? Because some signs are highly iconic, researchers often assume that movements in these signs have the same properties as speakers’ gestures. To test this assumption, we compared spontaneous hand gestures produced by hearing speakers when they talk (co-speech gesture) to productive iconic hand signs produced by deaf signers when the signs superficially resemble co-speech gestures (classifier signs). We used motion tracking and kinematic analyses to disentangle the spatial and temporal kinematic patterns of communicative movements in 33 English-speakers and 10 American Sign Language (ASL) signers, using each group’s non-communicative movements as a control. Participants copied a movement on an object performed by a model (non-communicative movement) and then described what they did with the object (communicative movement). We found no differences between groups in how non-communicative movements related to communicative movements for spatial kinematics. However, for temporal kinematics, speakers’ co-speech movements were *less* rhythmic and jerkier than their non-communicative movements, but signers’ communicative movements were *more* rhythmic and smoother than their non-communicative movements. We thus found differences in the temporal aspects of co-speech gestures vs. classifier signs, leading to 3 conclusions: (i) Communicative movements do not always have the same kinematic signatures but depend on the expressive system within which they are embedded. (ii) Since signers’ and speakers’ communicative movements have different kinematic features, even highly iconic signed movements cannot be considered entirely gestural. (iii) We need fine-grained techniques to measure communicative movements, particularly when trying to identify the gestural aspects of sign. Communicative movements, even when superficially similar, differ as a function of the system they are part of.

## INTRODUCTION

There are systematic differences between a movement used to pick up a teacup (a *non-communicative* movement) and a movement used to tell someone how to pick up the teacup (*a communicative* movement) (Gärdenfors, 2017; Jacob & Jeannerod, 2005; Kirsh & Maglio, 1994). An emerging joint field in movement science and cognitive science asks how the pressure to communicate (which is often understood as a high-level cognitive demand) interacts with lower-level sensorimotor control processes responsible for non-communicative action (Pezzulo et al., 2019; Tomasello et al., 2005; Toni et al., 2008; Torricelli et al., 2023). Motion tracking and kinematic analysis are the tools this new field uses to address the question. The kinematic signatures of communicative movements contain a complex set of spatial and temporal features that differ from the kinematic signatures of non-communicative movements. For example, communicative movements are reported to use more space and more exaggerated trajectories (Becchio et al., 2008; Krishnan-Barman et al., 2017; Liu et al., 2022); to contain more parsed units and be more complex (Trujillo et al., 2021); and to have higher peak velocity (Becchio et al., 2008; Krishnan-Barman et al., 2017; Trujillo et al., 2018; Vesper & Richardson, 2014) than non-communicative movements.

Finding relatively stable kinematic differences between communicative movements and non-communicative movements is not surprising given that human communicators are likely to use species-specific kinematic modulations to capture the attention of their human listeners. Many communicative contexts do, in fact, involve making movement more visually salient than non-communicative contexts (Veale et al., 2017). Here, we explore the breadth of this phenomenon by examining whether the language modality within which a communicative movement is embedded influences the kinematics of that movement. We ask whether hearing speakers’ communicative co-speech gestures differ from their non-communicative actions in the same way that deaf signers’ communicative iconic signs differ from their non-communicative actions.

We might expect differences between hearing speakers’ gestural movements and deaf signers’ movements because sign languages of the deaf (e.g., American Sign Language, ASL; British Sign Language) are conventionalized linguistic systems. Since the manual modality bears the full burden of communication in sign, movements are conventionalized and highly routinized by frequent use. In contrast, co-speech gestures are spontaneously created during talk and do not conform to a conventional system of signs (although they are synchronized with the speech they accompany; Kendon, 1980; McNeill, 1992).

There is, however, a set of signs, called classifier signs, that are highly iconic and look similar to the gestures of hearing people. The individual components of a classifier sign (handshape, movement, location) each expresses a meaning, and those meanings combine to create the compositional meaning of the sign (Emmorey, 2003; Supalla, 1982). Although handshape in classifier signs is generally agreed to adhere to a constrained set of categories (Emmorey & Herzig, 2003), movement is not (Brentari & Padden, 2001)—movement in classifier signs is often assumed to be gestural (Liddell, 2000; Schembri, 2003). Evidence for this assumption comes from studies comparing movements that signers produce in classifiers when describing an event, to movements that speakers produce when describing the same event with their hands and no speech (i.e., silent gesture) (Schembri et al., 2005; Singleton et al., 1993). These studies found that signers’ and speakers’ movements were indistinguishable.

However, Goldin-Meadow and Brentari (2017) argue that it is premature to conclude that movement in classifier signs is gestural because previous studies have all used the same, traditional methods to describe movement. We might find differences in the movements used in classifier signs and gestures if we use fine-grained kinematic analyses rather than the naked eye (Namboodiripad et al., 2016; Pouw & Dixon, 2019; Sato et al., 2020; Trujillo et al., 2018). We hypothesize that the kinematics of communicative movements are modulated by the expressive system within which the movements are embedded. If so, an iconic movement might have different properties if used as a co-speech gesture vs. a classifier sign, even if the two are superficially similar.

To test this hypothesis, we use motion tracking and kinematic analysis to make fine-grained measurements of non-communicative and communicative movements in signers and speakers. The data we analyze come from the Describe task (see Methods) of a previously reported study of 33 English speakers and 10 ASL signers (Brown et al., 2021) (the study focused on handshape and did not analyze movement). All participants watched a videotape of a model moving a disk along a path. They were then asked (i) to move a stick^1^ along the same path (*non-communicative condition*) and then (ii) to describe the movements they made in speech (English speakers) or sign (ASL signers) so that another person would be able to reproduce the movements as accurately as possible (*communicative condition*). See Supplementary for video examples of a signer and a speaker performing the communicative task. The English-speakers were told to gesture during their descriptions; the signers naturally produced sentences that contained classifier signs in this task.

## METHODS

### Participants

Forty-five adults (19~68 years-old, 23 female) gave informed consent (University of Chicago Institutional Review Board Protocol #14-0765) and participated in the experiment. Two groups of adults were recruited: 33 native English speakers, and 12 Deaf native ASL signers or early learners who learned ASL before six years old. Movement trajectories were not extracted from two signers because they did not use classifier predicates when doing the task; thus, only 10 signers were included in the current study. The sample size was determined in the previous study (Brown et al., 2021) by effect and sample sizes from the literature on visual-haptic illusions and by pilot data. For the current study, the data set constitutes a convenience-sized sample for exploratory purposes. As Deaf participants who learned ASL early in life are difficult to locate, the current dataset contains a smaller number of signers than speakers. To compensate for the small sample size of Deaf native ASL signers, multiple trials were taken in each experimental condition. All participants self-reported normal or corrected-to-normal vision and were confirmed to be right-handed using the Edinburgh Handedness Inventory (Oldfield, 1971).

### Task and Procedure

The current study is nested within a larger project designed to examine how ASL signers and English speakers Act on objects of various sizes, Estimate the lengths of the objects, and Describe how they moved the objects (Brown et al., 2021). The current study uses data from the Describe task of this larger project. In each trial, participants sat at a table and placed their right fist on a mark on the table (the home point), holding their thumb and forefinger extended and pressed together at the fingertips. Participants were asked to close their eyes while the experimenter placed a stick on the top of a piece of background paper. The participants were then instructed to open their eyes and to do three tasks, whose order was counterbalanced throughout the experiment: Action, Estimation, Describe. In the Describe task speakers and signers watched a video of a hand moving a disk along a path and were asked to re-enact the movement (*non-communicative condition*). They picked up the stick, holding it at its ends, and moved it along the modeled trajectory; they then placed the stick back in its original position and returned their hand to the starting point. They were then asked to describe what they did with the stick (*communicative condition*). Participants were told that their descriptions should be accurate enough that they could be shown to another person who would be asked to reproduce the movement. Each participant completed the procedure twice over 2 days. Examples from the video recordings of one data point for the same item by a speaker and signer can be found in the Supplementary Materials.

### Motion Tracking and Kinematic Data Preprocessing

Movements generated by the participants were recorded by a Phasespace Motion Capture system (sampling rate = 480 Hz, camera resolution = 3600 × 3600 pixel), including eight motion-capture cameras mounted on the walls of the testing room to record fine motor input from infrared-light emitting diode markers placed on the body. Two markers were placed on the thumb and index finger of the right hand and one marker was placed on the back of the hand at the wrist (for reconstructing grasping gestures; Brown et al., 2021). Wires extending from the sensors were secured on the arm to avoid any impediment to natural movement. Our analysis focused on the gross movement trajectories of the hand during the moving phase; we included the kinematic data recorded from the marker on the right index finger. 6174 movement trajectories were captured in total (signers: 759, 761; speakers: 2322, 2332; for the non-communicative and communicative conditions, respectively). The data were first smoothed using a fourth order zero-lag low-pass Butterworth filter with a resampling frequency of 120 Hz. We then excluded trajectories that contained more than 12 successive missing samples (i.e., 100 milliseconds of recording). To minimize the impact of recording error and to ensure that participants engaged in the moving tasks, we examined the distribution of displacement of all trajectories within each participant, and we excluded the trajectories whose maximal displacement was shorter than the mean minus 3 standard deviations of the distribution. To balance the number of trajectories between task conditions, the detected outlier in one condition, as well as its counterpart in another condition, were excluded together (note that this practice, necessary to balance the design, had the effect of doubling the exclusion rate). These criteria led to 5070 trajectories in our final analysis (signers: 681, 681; speakers: 1854, 1854; for the non-communicative and communicative conditions, respectively).

### Kinematic Analyses

Unlike classic manual classification and qualitative analysis of videos, in the current study we apply motion analysis (Namboodiripad et al., 2016; Pouw & Dixon, 2019; Sato et al., 2020; Trujillo et al., 2018) to measure subtle kinematic features of signs and gestures in space and time.

#### Spatial Features.

We assessed two spatial features. The *maximal covered area* was calculated as the area under the maximal *x* and *y* coordinates (horizontal to the table) of the trajectory (given in mm^2^). The dissimilarity of the trajectory shapes in non-communicative vs. communicative trajectories was calculated as the *Dynamic Time Warping (DTW) distance*. The DTW algorithm performed a non-linear temporal transformation while maintaining ordering of a query time-series (communicative trajectory) to seek an optimal alignment of a reference time-series (non-communicative trajectory). The resulting error (Euclidean distance) reflects the spatial dissimilarity in the comparison between the two time-series (Müller, 2007; Pouw & Dixon, 2019). The DTW performed was a dependent multivariate variable, and the distances reported were normalized to the length of the time-series. We conducted the analysis with the R package *dtw* (Giorgino, 2009).

#### Temporal Features.

From the same DTW analysis, we extracted a temporal feature of the movements, the *DTW warping cost*. This measure quantifies the extent to which the communicative trajectory is temporally warped to match the non-communicative trajectory in space, computed as the mean difference of sample indices between the warped communicative trajectory and the non-communicative trajectory. A positive value indicates that the warped communicative trajectory was more stretched temporally than the non-communicative trajectory; a negative value indicates that the warped communicative trajectory was more compressed temporally than the non-communicative trajectory.

Additional kinematic features were chosen to qualify, in a multidimensional way, possible changes in non-communicative vs. communicative trajectories including jerkiness, sample entropy, and rhythmicity of strokes and holds. Informed by our previous work (Pouw et al., 2021), we examined kinematic features that have been found to change in evolving silent gesture languages in iterated learning experiments. To extract these kinematic features, we need a lower-dimensional trajectory to capture the gross movements; we therefore calculated the 2D speed of the movements (smoothed by a Kolmogorov-Zurbenko filter with a smoothing window of 2, and 3 iterations).

*Jerkiness* indicates the smoothness of a movement—the higher the jerkiness score, the less smooth the movement. This measure computes changes that differ from a baseline, including changes in speed and breaks in motion. Here, jerkiness was formulated as dimensionless squared movement jerk (*x*‴), integrated over time, then scaled to the maximum movement squared speed (v) and cubed duration (D) (Equation 1) (Hogan & Sternad, 2009). Since this measure was long-tailed, we log-transformed it.

$$\text{Jerkiness}=\int_{t2}^{t1}x^{\prime \prime \prime }{\left(t\right)}^{2}dt*\frac{D^{3}}{\text{max}\left(v^{2}\right)}$$ (1)

*Sample entropy* is an index of predictability of the speed time-series (Richman et al., 2004), which is an information theory-based measure of how often different patterns recur in a signal. This Shannon information is designed for time series analysis and is a commonly used measure for characterizing the structure of communicative signals (Motamedi et al., 2019; Verhoef et al., 2016). Higher values indicate more sample entropy in the signal and more unpredictability; lower values indicate more regularity and less unpredictability. Sample entropy was calculated as the logarithmic probability that a pattern of length 2 remains similar in the next incremental comparison with time delay of 1; the similarity was assigned a threshold at 0.2 times the standard deviation of the signal. We compute sample entropy using the R package *pracma* (Borchers, 2023).

*Rhythmicity of strokes* and *rhythmicity of holds* are two related aspects of the rhythmicity of a trajectory. A sub-movement or ‘stroke’ was captured using a peak-finding function to identify and count the maxima in a given speed time-series (Pouw et al., 2021). We defined breaks between the sub-movements or ‘holds’ as having speed lower than 15 meters per second. Rhythmicity was calculated as the standard deviation of the temporal interval of strokes and holds. For clarity of interpretation, we reversed the signs of these measures so that a higher score indicates a more isochronous pattern and therefore greater rhythmicity. Since the two measures were long-tailed, we log-transformed them.

#### Statistical Inference.

Following the procedure of maximal random-effect structure (Barr et al., 2013), we used linear mixed models to account for unbalanced data between speakers and signers, and random variances over participants as well as test items (i.e., types of movements shown in the videos). For the outcome variables of DTW normalized distance and warping cost, we included one fixed effect of language group (signer/speaker), and two random intercepts for participants and test items (the random slope for language group that varied over items was removed for model convergence). For the other outcome variables, we modeled language group, movement condition (non-communicative/communicative), and their interaction as fixed effects. We started with the maximal models, which included all possible random effects and the grouping variables, participants and items. To resolve the model convergence issue, we simplified the models of maximal coverage and rhythmicity of strokes. For maximal coverage, we were only able to model the random slope of movement conditions over participants and two random intercepts as random effects. For rhythmicity of strokes, the random slope of language group and movement condition covariance over items was removed. The models were fitted with *lmer()* function in the *lme4* package (Bates et al., 2015). The model parameters were estimated using restricted maximum likelihood, and *p*-values were computed by *t*-test with Kenward-Roger approximation for denominator degrees of freedom. Post-hoc comparisons which focused on the interaction effects were conducted with *emmeans* (Lenth et al., 2021). All *p*-values were adjusted with Bonferroni correction for multiple comparisons.

## RESULTS

In each trial, participants were required to start moving from a fixed home point and return to the home point after each task. Kinematic recordings of the participant’s index finger were used to extract spatial and temporal features from the participants’ non-communicative movements and communicative movements (see Table S.1 in Supplementary Materials for measures and analyses of additional kinematic features: peak velocity, acceleration, movement duration, movement height).

### Speakers and Signers Maintain Spatial Features Across Non-Communicative and Communicative Movements

**Figure 1.**
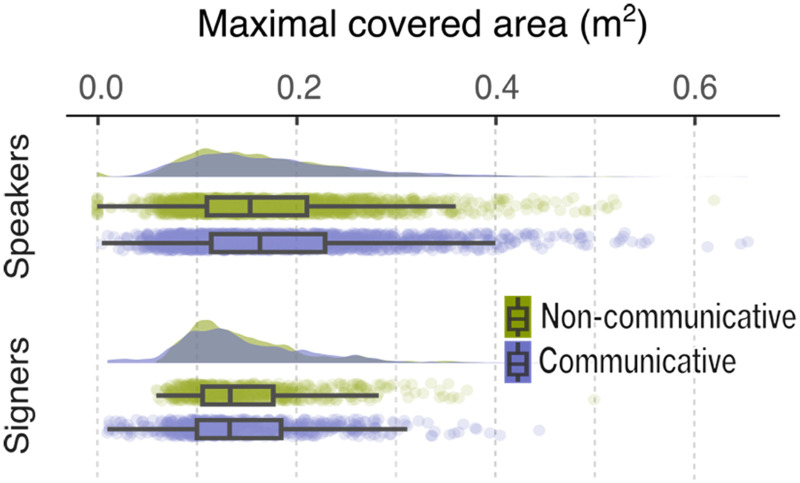
**Maximal area covered under the moving trajectories.** The curved areas illustrate kernel density estimates of the data distribution. The colored dots are jittered raw data. The boxes indicate the interquartile range (IQR) and the whiskers are maximum and minimum with 1.5 IQR. The black bars inside the boxes represent the mean value per condition. The non-communicative and communicative conditions are color coded with green and purple, respectively.

We first examined the maximal area covered by the movements generated by speakers and signers in the non-communicative and communicative conditions. We did not find statistically significant main effects of movement type (non-communicative, communicative) or language group (speakers, signers), nor did we find an interaction between the two (Figure 1 and Table 1). These results indicate that the speakers and signers covered comparable spaces in non-communicative and communicative movements.

**Table 1.** Main effects of language group (speakers, signers) and movement type (communicative, non-communicative), and their interactions, on kinematic features.

Kinematic feature	Effect	Estimate	*SE*	95% CI	*t*	kr-df	*p*_corr_
Maximal covered area	Language group	.04	.02	[.10, .18]	1.97	40.26	.42
	Movement type	.00	.01	[−.00, .08]	.41	37.47	4.77
	Interaction	−.02	.01	[−.02, .02]]	−1.38	38.05	1.24
Jerkiness	Language group	4.58	.56	[3.45, 5.70]	8.21	40.61	**<.001**
	Movement type	1.16	.41	[.34, 1.99]	2.87	39.98	.07
	Interaction	−4.73	.47	[−5.67, −3.78]	−10.15	40.57	**<.001**
Sample entropy	Language group	−.09	.01	[−.11, −.07]	−8.72	41.08	**<.001**
	Movement type	−.02	.01	[−.05, .00]	−1.76	39.69	.63
	Interaction	.01	.02	[.07, .14]	6.64	40.53	**<.001**
Rhythmicity of strokes	Language group	−1.07	.20	[−1.48, −.66]	−5.28	40.84	**<.001**
	Movement type	−.13	.16	[−.46, .20]	−.79	40.70	3.01
	Interaction	1.13	.19	[.76, 1.51]	6.05	40.24	**<.001**
Rhythmicity of holds	Language group	−1.34	.24	[−1.84, −.85]	−5.52	41.22	**<.001**
	Movement type	−.09	.20	[−.48, .31]	−.44	39.87	.66
	Interaction	1.37	.23	[.92, 1.83]	6.07	40.83	**<.001**
DTW distance	Language group	−20.91	16.52	[−54.31, 12.48]	−1.27	40.15	1.49
DTW warping cost	Language group	927.54	155.76	[612.84, 1242.24]	5.95	40.45	**<.001**

The speakers and signers might, however, be carving out different shapes within this surface area. But the DTW distances between communicative vs. non-communicative trajectories did *not* differ statistically for speakers or signers (Figure 3C, *t*_40.15_ = −1.27, *p*_corr_ = 1.49, Estimate(*SE*) = −20.91(16.52) , 95% CI = [−54.41, 12.48]). The results suggest that the speakers and signers systematically organized the shapes of their communicative vs. non-communicative trajectories in similar ways.

To summarize thus far, the spatial characteristics of the movements that speakers and signers produced to reproduce a movement (non-communicative movements) were like those that both groups produced to describe the movement (communicative movements).

### Communicative Movements in Speakers’ Gestures Are Temporally Stretched Relative to Their Non-Communicative Movements; Signers’ Movements Are Compressed

**Figure 2.**
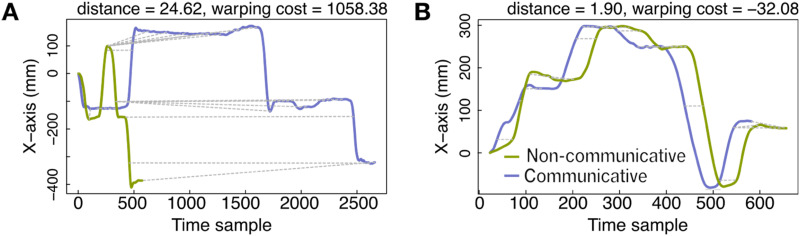
**Dynamic Time Warping (DTW) analysis.** Distance (reflecting spatial properties) and warping cost (reflecting temporal properties) are two measures in DTW analysis. **A–B** each presents an example of an individual’s trajectory in the non-communicative condition (in green) compared to the same individual’s trajectory in the communicative condition (in purple). In **A**, we achieve spatial alignment between the non-communicative and communicative trajectories by temporally stretching the communicative trajectory to fit the non-communicative trajectory; the stretch is relatively large, resulting in a large warping cost. In **B**, we achieve spatial alignment by temporally compressing the communicative trajectory to fit the non-communicative trajectory; the compression is relatively small, resulting in a small warping cost.

To explore the temporal features of the participants’ movements, we first examine the warping cost of the DTW procedure. The warping cost of the DTW procedure tells us the relative temporal warping between the query (communicative movement) and referent (non-communicative movement) trajectories. To illustrate, we plot pairs of non-communicative (green) and communicative (purple) trajectories generated within one trial in Figure 2A and another trial in Figure 2B. The *x*-axis represents time samples, and the *y*-axis the participant’s finger position on the horizontal axis along the table^2^. The shapes of the non-communicative and communicative movement trajectories in Figure 2A and 2B are similar, but the extent to which the trajectories had to be temporally warped to overlay the communicative trajectory onto the non-communicative trajectory differ. Figure 2A illustrates a large warping cost (a stretched communicative trajectory relative to the non-communicative trajectory); Figure 2B illustrates a small warping cost (a compressed communicative trajectory relative to the non-communicative trajectory).

**Figure 3.**
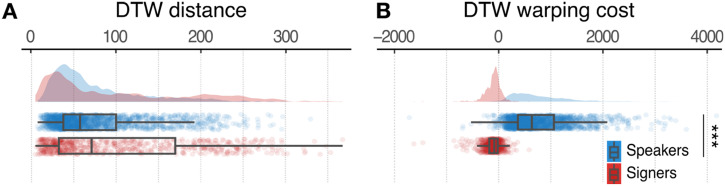
**Composite results of DTW analysis.** **A** summarizes the spatial properties of the trajectories and shows little difference between speakers (in blue) and signers (in red). **B** summarizes the temporal properties of the trajectories and shows no overlap between the distributions for speakers and signers. The curved areas illustrate kernel density estimates of the data distribution. The colored dots are jittered raw data. The boxes indicate the interquartile range (IQR) and the whiskers are maximum and minimum with 1.5 IQR. The black bars inside the boxes represent the mean value per condition ***: *p*_corr_ < .001.

We applied this analysis to all of the communicative/non-communicative comparisons for speakers and signers and found that the DTW warping costs patterned differently for the two groups of participants (*t*_40.45_ = 5.95, *p*_corr_ < .01, Estimate(*SE*) = 927.54(155.76), 95% CI = [612.84, 1242.24]). Unlike the DTW distance data, which were comparable for speakers and signers (Figure 3A), DTW warping costs differed for the two groups (Figure 3B). Warping costs were significantly higher than zero for speakers (Mean(*SE*) = 772.86(13.18), *t*_1853_ = 58.676, *p*_corr_ < .001, 95% CI = [747.03, 798.69]), but significantly lower than zero for signers (Mean(*SE*) = −110.66(6.23), *t*_680_ = −16.71, *p*_corr_ < .001, 95% CI = [−123.67, −97.65]). In other words, speakers produced trajectories in the communicative condition that were more temporally *stretched* than the trajectories they produced in the non-communicative condition. In contrast, signers produced trajectories in the communicative condition that were more temporally *compressed* than trajectories in the non-communicative condition. The underlying temporal compression and dilation captured by DTW are unlikely to be linear or uniform, but rather intermittent and variable. These results suggest that speakers and signers differ in their control of the temporal aspects of the movements they produce in a communicative task. We focused on four temporal kinematic features to examine the temporal organization of the movements in more detail.

### Speakers’ Communicative Movements Are Less Smooth, Less Rhythmic, and Less Predictable Than Their Non-Communicative Movements; Signers’ Movements Display the Opposite Pattern

Four temporal kinematic properties were quantified and analyzed to better understand the relationship between non-communicative and communicative movements in speakers and signers: jerkiness, sample entropy and rhythmicity of strokes and holds.

#### Jerkiness.

**Figure 4.**
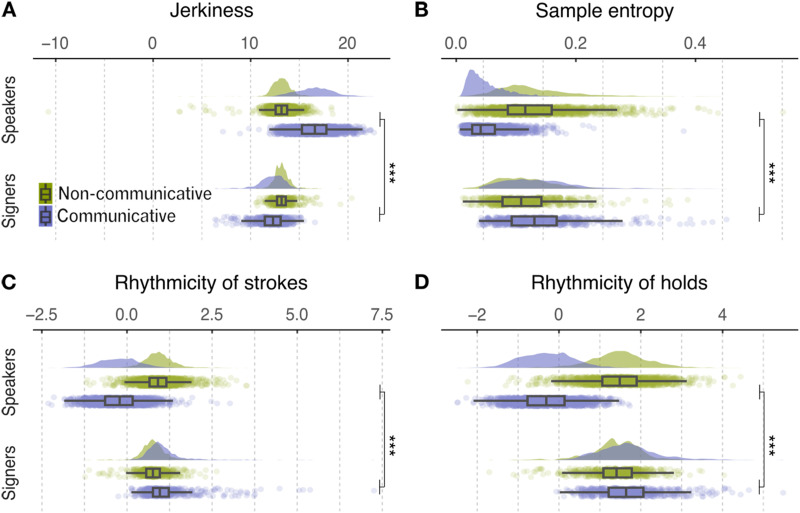
**Speakers and signers showed reversed patterns in Communicative movement versus Non-communicative movement conditions with respect to jerkiness (A) sample entropy, (B) rhythmicity of strokes (C), or rhythmicity of holds (D) in the movement trajectories.** The curved areas illustrate kernel density estimates of the data distribution. The colored dots are jittered raw data. The boxes indicate the interquartile range (IQR) and the whiskers are maximum and minimum with 1.5 IQR. The black bars inside the boxes represent the mean value per condition. The communicative and non-communicative conditions are color coded with green and purple, respectively. ***: *p*_corr_ < .001.

We found a significant interaction between language group and movement condition for jerkiness (*t*_40.57_ = −10.15, *p*_corr_ < .001, Estimate(*SE*) = −4.73(.47), 95% CI = [−5.67, −3.78], Figure 4A). For speakers, jerkiness was higher in the communicative condition than in the non-communicative condition (*t*_39.7_ = 15.58, *p*_corr_ < .001; Means(*SE*s) = 16.7(.27) & 13.1(.16), 95% CIs = [16.1, 17.2] & [12.8 13.4], for communicative and non-communicative, respectively). For signers, jerkiness showed the reverse effect (*t*_37.1_ = −2.87, *p*_corr_ = .01; Means(*SE*s) = 12.1(.49) & 13.2(.27), 95% CIs = [11.1, 13.0] & [12.7, 13.8]). In other words, speakers’ communicative movements were more jerky (less smooth) than their non-communicative movements. In contrast, signers’ communicative movements were less jerky than their non-communicative movements.

#### Sample Entropy.

We found a significant interaction between language group and movement condition for sample entropy of the movement (*t*_40.53_ = 6.64, *p*_corr_ < .001, Estimate(*SE*) = .10(.02), 95% CI = [.07, .14], Figure 4B). Specifically, in speakers, communicative movements were less predictable than non-communicative movements (*t*_44.8_ = −10.12, *p*_corr_ < .001; Means(*SE*s) = .05(.01) & .13(.01), 95% CIs = [.04, .06] & [.11, .16]). In contrast, in signers, communicative movements were marginally more predictable than in the non-communicative movements; this effect did not survive the correction for multiple comparisons (*t*_38.6_ = 1.76, *p*_corr_ = .23; Means(*SE*s) = .14(.01) & .11(.01), 95% CIs = [.12, .16] & [.09, .14]. In other words, speakers produced less predictable communicative movements than non-communicative movements. Signers used the same level of sample entropy when producing communicative and non-communicative movements.

#### Rhythmicity of Strokes and Holds.

Finally, we assessed rhythmicity in terms of strokes and holds. We found a significant interaction between language group and movement condition for both rhythmicity of strokes (*t*_39.56_ = 10.83, *p*_corr_ < .001, Estimate(*SE*) = 1.47(.14), 95% CI = [1.19, 1.74], Figure 4C) and rhythmicity of holds (*t*_40.15_ = 12.60, *p*_corr_ < .001, Estimate(*SE*) = 2.08(.16), 95% CI = [1.74, 2.41], Figure 4D). Post-hoc analysis showed that, for speakers, both rhythmicity of strokes (*t*_40.2_ = −16.89, *p*_corr_ < .001; Means(*SE*s) = −.20(.07) & .92(.04), 95% CIs = [−.34, −.05] & [.84, 1.01]) and rhythmicity of holds (*t*_40.7_ = −21.93, *p*_corr_ < .001; Means(*SE*s) = −.26(.08) & 1.51(.08), 95% CIs = [−.43, −.10] & [1.37, 1.66]) were systematically lower in the communicative condition than in the non-communicative condition. In contrast, for signers, rhythmicity of strokes was systematically higher in the communicative condition than in the non-communicative condition (*t*_39.0_ = 2.96, *p*_corr_ = .01; Means(*SE*s) = 1.13(.13) & .78(.08), 95% CIs = [.87, 1.38] & [.63, .92]), as was rhythmicity of holds after correction for multiple comparisons(*t*_38.6_ = 2.09, *p*_corr_ = .11; Means(*SE*s) = 1.68(.15) & 1.38(.13), 95% CIs = [1.39, 1.98] & [1.13, 1.64]). Movements that speakers used in their descriptions were less rhythmic than movements they used to act on an object. In contrast, movements that signers used in their descriptions were more rhythmic than movements they used to act on an object.

Importantly (and not surprisingly), we did not find significant between-group differences in any of these kinematic features in the non-communicative condition (Table 2). In other words, speakers and signers were comparable in how they controlled their movements when there were no communicative demands.

**Table 2.** Simple effect of language group on kinematic features of non-communicative movements.

Feature	Mean		*SE*		95% CI		*t*	df	*p*_corr_
	Speaker	Signer	Speaker	Signer	Speaker	Signer			
Jerkiness	13.1	13.2	.16	.27	[12.8, 13.4]	[11.1, 13.0]	.48	38.2	7.00
Sample entropy	.13	.11	.00	.01	[.12, .15]	[.09, .14]	−1.09	40.0	5.96
Rhythmicity of strokes	.92	.78	.04	.08	[.83, 1.01]	[.62, .93]	−16.90	38.8	2.10
Rhythmicity of holds	1.51	1.38	.08	.13	[1.36, 1.67]	[1.12, 1.65]	−.87	38.4	7.00

## DISCUSSION

In this study, we analyzed how speakers and signers organize their movements for communicative purposes, and we compared those movements to the movements they used for non-communicative purposes. Participants were asked to reproduce a movement on an object (non-communicative), and then describe their movement so that someone else could perform it (communicative). This procedure allowed us to assess, within an individual, the kinematics of movements designed to either mimic an action on an object or communicate about the action. The choice of participants (English-speakers, ASL-signers) allowed us to assess the impact of different systems of communication on the kinematics of movement. Sign languages are linguistic systems; gestures that accompany speech are spontaneously produced and vary as a function of the spoken language they accompany (Gullberg, 2003, 2010; Kita & Özyürek, 2003; McNeill & Duncan, 2000).

We found that both speakers and signers displayed the same spatial features in their communicative movements, compared to their non-communicative movements. We also found that both groups modified the temporal features of their communicative movement trajectories relative to their non-communicative movement trajectories, but in opposite directions. Not only were the speakers’ co-speech gestures in the communicative condition grossly stretched in time, but they were also less smooth, less rhythmic and less predictable than their movements in the non-communicative condition. In contrast, the signers’ movements in the communicative condition were compressed in time, and also smoother and more rhythmic than their movements in the non-communicative condition. Earlier studies have shown relatively stable findings in how communicative movements differ from non-communicative movements (more space, higher velocity, more multi-unit trajectories etc.) (Becchio et al., 2008, 2010; Krishnan-Barman et al., 2017; Liu et al., 2022; Trujillo et al., 2018, 2021; Vesper & Richardson, 2014). However, our results indicate that the way communicators vary their movements relative to their non-communicative movements is not always the same—their communicative movements depend on the expressive system within which they are embedded.

Our findings underscore the fact that sign language, including classifier constructions, displays the hallmarks of efficient, compressed, and resilient communication in the visual modality. Critically, our report is also a first important step in understanding how movement in classifier signs and co-speech gestures differ from one another. Communicative movements vs. non-communicative movements patterned in different ways for speakers and signers. Why would signers’ communicative movements be smoother and more rhythmic than their non-communicative movements, and why would speakers’ communicative movements be more jerky, less rhythmic and less predictable than their non-communicative movements? We consider several explanations for these differences in temporal kinematics.

First, the temporal differences between signers’ and speakers’ communicative movements could reflect the fact that signers may be more aware than speakers of the impact that their movements have on their recipients. Signers may produce smoother movements when they are communicating because smooth movements are more predictable (and thus more interpretable) than less smooth movements (Vesper et al., 2011). Signers also increase rhythmicity in their communicative movements relative to their non-communicative movements, which could serve as an ostensive cue to attract the attention of the interacting partner (since it deviates from the usual way of moving and thus might attract attention) (Csibra, 2010; Sperber & Wilson, 2024; Tomasello & Kruger, 1992). In contrast, speakers often do not realize that they are gesturing when they speak (Goldin-Meadow, 2003) and therefore rarely think about the effect of their gestures on their listeners. Note, however, that this hypothesis cannot account for the fact that speakers’ gestures are less smooth than their non-communicative movements, nor for the fact that the two groups’ movements vary in rhythmicity in opposite ways. Speakers’ gestures might also be expected to be *more* rhythmic than their non-communicative movements since the rhythmicity effect has been found in a variety of communication situations, including multimodal communication.

Second, the temporal differences between signers and speakers might reflect the fact that their communicative movements need to be incorporated into different linguistic systems (Chu & Hagoort, 2014; Wagner et al., 2014). Signers rely on a conventional manual system to communicate, and their classifier signs must be integrated into the sign stream, which imposes different constraints from those imposed by the speech stream. In ASL, classifier signs are incorporated into the structure and prosody of the signed sentences as word units. Even the relatively free-formed movements in classifier signs are bound by the conventionalized routines used in lexical signs (Ferrara & Napoli, 2019; Napoli & Ferrara, 2021). In contrast, the co-speech gestures accompanying English are incorporated into the structure of the spoken sentences of the phrasal unit and further aligned to prosody (Fenlon et al., 2019; Loehr, 2004).

Along these lines, for spoken language, Torre et al. (2019) show that physical instantiations of speech comply with linguistic laws, indicating that signal compression is a typical characteristic of a high-density information transfer system. For sign language, Andres et al. (2021; see also Borneman et al., 2018; Malaia et al., 2018) find the same pattern in Czech Sign Language, and we also find compression in time in our sign data, suggesting that efficiency constraints underlying language structure are not modality specific. However, co-speech gesture is not a contained linguistic system. As a result, it is not likely to be constrained by the same principles of communicative efficiency as speech or sign, nor is it likely to have evolved toward signal compression. The relation between co-speech gesture and communicative efficiency needs to be explored empirically.

Third, the cost of coordinating and integrating gesture into speech could explain the differences in temporal kinematics between speakers and signers. When organizing their gestures in relation to speech, speakers need to coordinate vocal-articulatory movements with their hand movements (Kendon, 2004; Loehr, 2004; McClave, 1998; McNeill, 1985, 1992). The jerkier communicative trajectories relative to non-communicative trajectories may be a product of the increased cost that multimodal demands impose on speakers’ motor control. Support for this hypothesis comes from our Shannon-based entropy calculations of sample entropy. For speakers, entropy decreases in communicative movements relative to non-communicative movements, suggesting that some information may be off-loaded onto the speech that accompanies the gestures. In contrast, signers do not have the option of off-loading information onto another modality, and there were no significant differences in entropy between communicative and non-communicative movements for signers.

In future work, we will analyze how the talk that accompanies communicative gestures interacts with the temporal kinematics of manual movements, applying information metrics to the speech that accompanies gesture. We hypothesize that speakers’ co-speech gestures may be longer in communicative contexts to accommodate the longer spoken phrasal units they accompany. We also hypothesize that these gestures will contain less information than signers’ classifiers since, for speakers, some of the information will be off-loaded onto speech. However, we predict that speech + gesture, taken together, will contain comparable amounts of information as the signer’s classifiers (see Goldin-Meadow & Brentari, 2017, for discussion of how sign and speech should be compared).

Our findings underscore the need for a more fine-grained movement science of gesture and sign. We know very little about basic natural statistics of sign versus gesture. Although we know that sign languages have slower oscillatory components than speech (Brookshire et al., 2017), we know little about the kinematic differences between signs and co-speech gestures. Similarly, we know a great deal about how co-speech gestures integrate kinematically with speech prosody (Loehr, 2004; Wagner et al., 2014), but much less about prosodically coordinated utterances in sign languages (Kimmelman et al., 2020). Future studies using our analytic approach are needed to explore the differences and similarities between gesture and sign in tasks that go beyond describing simple events in which an object is moved across space by hand.

## CONCLUSION

We have found that signers and speakers tune the temporal parameters of their communicative movements in opposite directions relative to their non-communicative movements. Even when signs are superficially comparable to spontaneously generated gestures, they have different temporal kinematics. This result calls into question the stability of kinematic properties across communication contexts. The findings also lend support to the claim that signers’ communicative movements are different from speakers’ communicative movements, suggesting that movement in signers’ classifiers, despite its high level of iconicity, is not entirely gestural.

## ACKNOWLEDGMENTS

We thank members of Visual Communication (ViCom) for helpful comments on the manuscript.

## FUNDING INFORMATION

This project was funded by a grant entitled The Body’s Role in Thinking, Performing and Referencing from the Neubauer Collegium for Culture and Society to the Center for the Study of Gesture Sign and Language at the University of Chicago. WP is supported by Nederlandse Organisatie voor Wetenschappelijk Onderzoek (NWO) and Deutsche Forschungsgemeinschaft (DFG). Artificial intelligence: No artificial intelligence assisted technologies were used in this research or the creation of this article.

## AUTHOR CONTRIBUTIONS

SGM, DB designed research; RL, WP analyzed data; RL, DB wrote the first draft of the manuscript, and all authors edited the manuscript.

## DATA AVAILABILITY STATEMENT

The data and code that support the findings of this study are available at osf.io/7nsaz.

## Notes

^1^ The current study is nested within a larger project comparing grip aperture of the right hand in ASL signers and English speakers in a variety of tasks (see Methods). Here, we focus not on handshape, but on motion, examining the trajectory of the right index fingertip.^2^ To simplify our illustration, we present only one-dimensional data; in the formal analysis, we conducted the DTW analysis in 2D space; see Methods.
